# Clinical application and developmental direction of free flaps in plastic and reconstructive surgery procedures: a bibliometric analysis

**DOI:** 10.3389/fsurg.2025.1661571

**Published:** 2025-11-19

**Authors:** Xinghao Yin, Qianqian Hua, Jiangtian Ye, Shangjing Xie, Ruitong Lv, Leyi Cai, Haixun Li

**Affiliations:** 1 Department of Orthopaedics Surgery, First People's Hospital of Yongkang City, Yongkang City, Zhejiang, China; 2 Department of Orthopaedics Surgery, The Second Affiliated Hospital and Yuying Children's Hospital of Wenzhou Medical University, The Second School of Medicine, Wenzhou Medical University, Wenzhou, Zhejiang, China; 3 Department of Orthopaedics, Shanghai Sixth People's Hospital Affiliated to Shanghai Jiao Tong University School of Medicine, Shanghai, China; 4 Department of Respiratory Medicine, Ruijin Hospital, Shanghai Jiaotong University School of Medicine, Shanghai, China; 5 Key Laboratory of Diagnosis and Treatment of Severe Hepato-Pancreatic Diseases of Zhejiang Province, The First Affiliated Hospital of Wenzhou Medical University, Wenzhou, Zhejiang, China

**Keywords:** free flap, plastic surgical procedures, bibliometric analysis, visualization, global trends

## Abstract

**Introduction:**

Advancements in microvascular surgical techniques have significantly improved the success rate of free flap transplants, making it a preferred method for repairing postoperative tissue defects caused by tumors, trauma, and infections. Despite numerous clinical studies on free flaps in wound reconstruction, comprehensive bibliometric analyses to systematically review their clinical applications and identify emerging research trends are lacking.

**Methods:**

This study analyzed literature from the Web of Science Core Collection (WOSCC), Pubmed, and Embase databases spanning 2004 to 2025. We examined annual publications, global collaborations, research frameworks, and identified emerging research trends and key keywords.

**Results:**

The analysis revealed that current research focuses on two core areas: the application of free flaps in reconstructive procedures and the emerging intersection of computer technology with medical and material research.

**Discussion:**

These findings provide valuable insights to guide future scholarly inquiries into the utilization of free flaps in plastic and reconstructive surgery procedures.

## 1 Introduction

Free flaps are defined as tissue segments whose blood supply from the donor site is completely detached, and are transplanted to the recipient site by establishing vascular connections using microsurgical methods (1). Advancements in clinical microvascular tissue transplantation and postoperative monitoring techniques have significantly improved the survival rates of free flap transplants, which now stand at 90%–98% (2). The application of free flaps has broadened, establishing them as a crucial strategy for repairing breast reconstruction (3), hand injuries (4), traumatic limb injuries (5), craniofacial reconstructions (6), burns (7), and complex abdominal wall defects (8). Flaps are now not only used to repair soft tissue defects but also to restore the natural cosmetic appearance and function of the trauma site to the greatest extent possible (9).

Bibliometric analysis gathers comprehensive literature and citation data across platforms, providing quantitative insights into publications within a field. It identifies the most influential authors, countries, journals, and institutions contributing significantly to the topic, offering a clear understanding of the field's structure and dynamics. This method enables conclusions to be drawn from the visual cues of graphs, such as color and size. Unlike traditional reviews, bibliometric analysis adopts a wider international view, minimizes subjective bias, and integrates historical with future research perspectives. Recent bibliometric studies have highlighted the critical role of vascularization in flap survival (10) and the potential of infrared thermography to monitor flap survival (10). Not only has there been innovation in terms of technology, but bibliometrics has also played a crucial role in guiding advancements in the survival of flaps, particularly in relation to drugs and mechanisms (11, 12).

Over the last two decades, scholarly articles have increasingly addressed the diversity of free flaps, anastomosis techniques, their clinical utility, and management of postoperative complications. Despite this growing body of literature, a comprehensive overview of the application of free flaps in plastic surgery is conspicuously absent, as are projections about the future trajectory of clinical use and research in this domain. This article aims to synthesize and visually depict the latest focal points and advancements in the field of free flaps in plastic surgery.

## 2 Materials and methods

### 2.1 Sources of bibliometric data and search strategy

We conducted a comprehensive search using the advanced search feature of the WOSCC database Pubmed database and Embase database to collate literature pertaining to free flaps in plastic surgery procedures.

**Figure 1:**
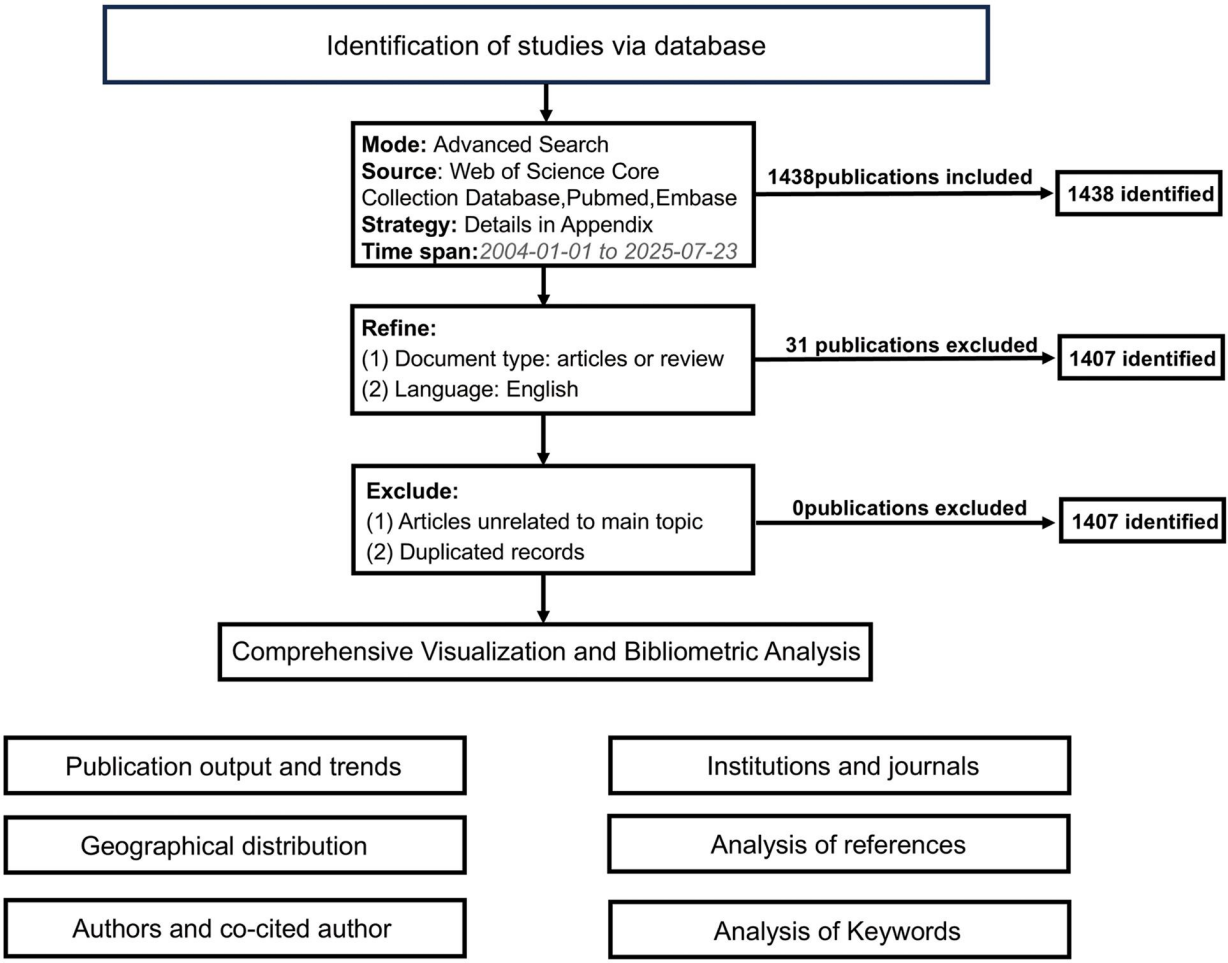
Flow chart of bibliometric analysis.

Our analysis was restricted to literature published from January 1, 2004 to July 23, 2025, identifying a total of 1,438 articles. We further refined the selection to include only “articles” or “reviews”. Utilizing CiteSpace software, we thoroughly reviewed titles and abstracts to eliminate any irrelevant or duplicate articles, resulting in a final tally of 1,407 articles. The complete record for each article was downloaded. The detailed methodology and search strategy is outlined in Figure 1 and Supplementary Figure S1.

### 2.2 Data extraction and analysis

For visualizing geographical distribution of publications, tools such as the Scimago Graphica (version 1.0.36) and Microsoft Charticulator (https://charticulator.com/) were employed. Besides, analysis of authors and co-cited authors, journals, institutions, references, and keywords mainly involved software and websites like VOS viewer (version 1.6.20) (13), bibliometrix (14), and Citespace (version 6.2.R6).

## 3 Results

### 3.1 Publication output and trends

**Figure 2:**
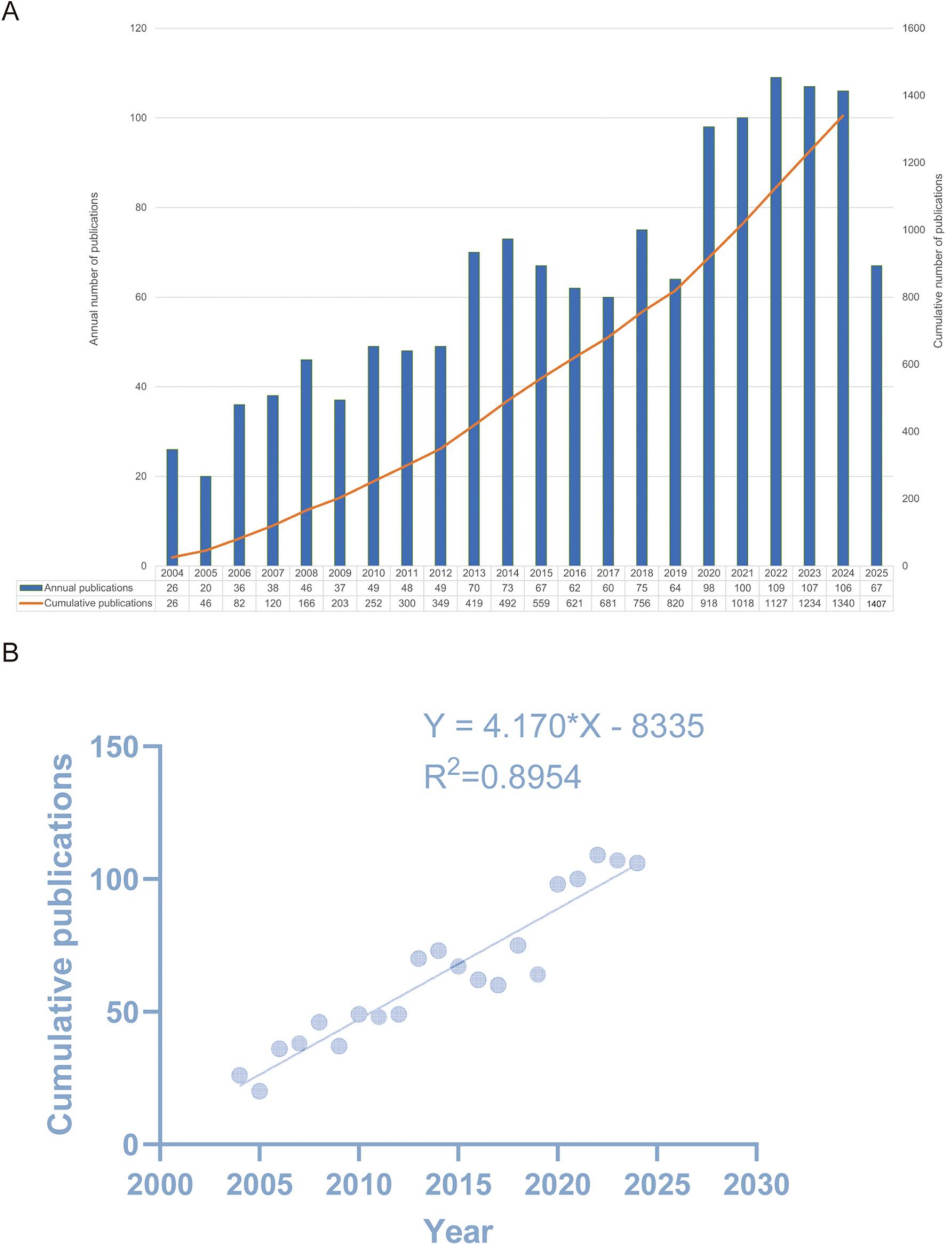
**(A)** Distribution of publication output and trends in the cumulative number of publications from 2004 to 2025. The blue bar graph represents the number of publications per year, and the solid orange line represents the cumulative number of publications. **(B)** Model fitting curves of global trends in publications (*R*^2^ = 0.895).

Figure 2A illustrates the publication trends, revealing a consistent upward trajectory in publication volume. In 2022, a total of 109 publications were recorded, representing a substantial increase—approximately three to four times the publication count in 2004—indicating a period of explosive growth from 2019 to 2025. To further assess the progress of research in this field, we excluded incomplete data for 2025 and performed a linear regression analysis using publication data from 2004 to 2024, as shown in Figure 2B. The results indicate a steady increase in the number of publications, with the fitted equation given by: y = 4.170x − 8335, *R*^2^ = 0.8954.

### 3.2 Geographical distribution of publications

**Figure 3:**
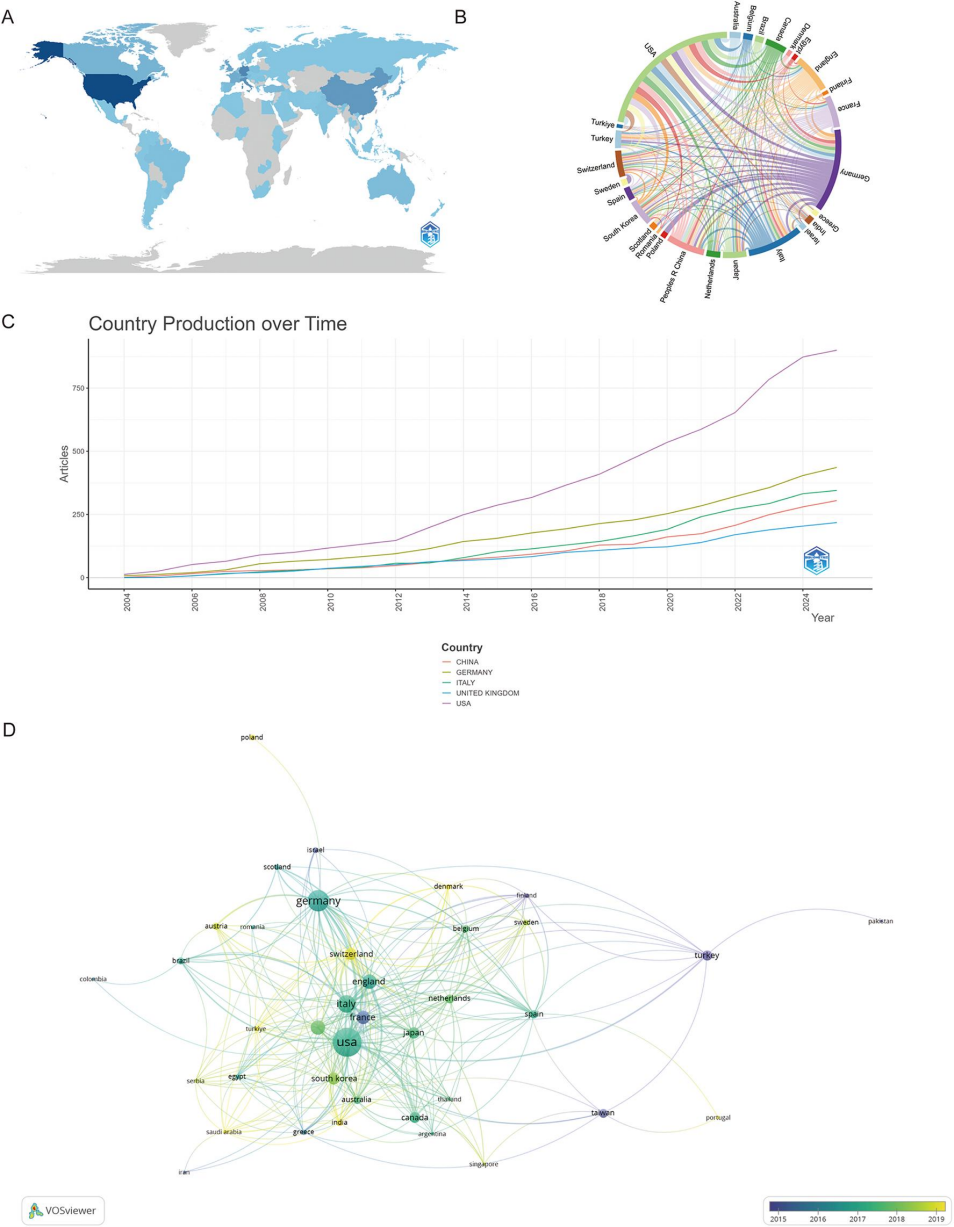
**(A)** The global distribution of publications is shown, with the circle size proportional to the number of publications for each country. **(B)** Analysis of international collaboration among different countries/regions is presented. Links between countries/regions represent cooperative relationships, with thicker lines indicating stronger collaborations. **(C)** Country production overtime. **(D)** An overlay visualization map of country analysis.

Research in this field involves contributions from 69 countries. Figure 3A illustrates the global distribution of publications in this domain, with darker blue shades indicating a higher volume of publications. To provide further detail, we consolidated the publication data from each country, with Table 1 enumerating the top 10 countries with the highest research output. The United States leads with 900 articles, accounting for 63.97% of the total publications, and holds the highest citation count at 6,318. Germany follows with 436 articles. As shown in Figure 3C, the steeper slopes of the purple, brown, and red lines suggest that the United States, Germany, and China are experiencing pronounced growth in publication numbers, highlighting their strong potential for future scientific contributions.

**Table 1:** The top 10 countries/regions with the greatest numbers of publications.

Country	Publications	*N* (%)	Total citation	Average article citations
USA	900	63.97%	6,318	19.90
Germany	436	30.99%	1,855	10.00
Italy	345	24.52%	2,675	22.70
China	305	21.68%	1,148	9.40
UK	218	15.49%	1,501	18.50
France	175	12.44%	1,613	24.40
Japan	140	9.95%	546	11.40
South Korea	127	9.03%	580	9.80
Canada	125	8.88%	631	15.00
Switzerland	120	8.53%	713	17.00

The chord diagram in Figure 3B illustrates the collaborative networks among countries, with Germany, the United States, Italy, and China being key players in international research partnerships. Lastly, Figure 3D reveals that France was one of the pioneering countries in this field, while Switzerland, through its close international collaborations, demonstrates significant scientific research potential.

### 3.3 Analysis of major institutions, core authors and co-cited author

**Figure 4:**
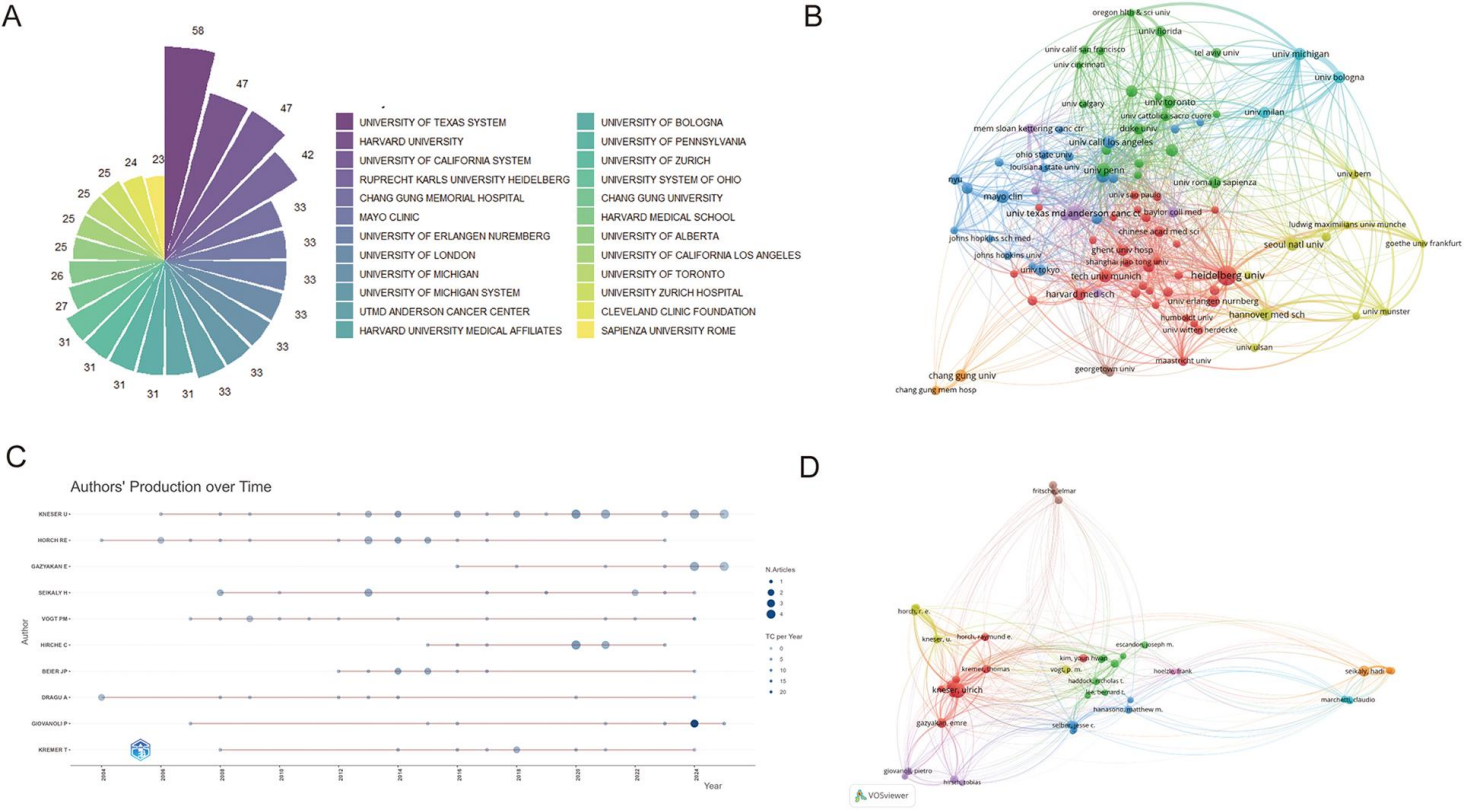
**(A)** The top 24 institutions with the most publications. **(B)** Network map of co-authorship between institutions with more than four publications. The scale of the circles is proportional to the institution's output, while the thickness of connecting lines signifies the intensity of collaboration, and color clusters denote the presence of cooperative relationships **(C)** The top 10 authors' production over time is shown. Circle size represents number of publications, with larger circles denoting more publications. **(D)** A visualization map exhibiting co-authorship. A minimum threshold of 30 citations per author was established, yielding 61 matches.

This study identified 1,175 institutions actively engaged in research within this field. As shown in Figure 4A, the top 24 contributing institutions are listed, with the University of Texas System leading with 58 publications. Ongoing monitoring of this institution's research output may provide valuable insights into emerging trends and directions in the field.

To further examine institutional collaboration, a co-authorship network map was generated based on total link strength (Figure 4B). The University of Michigan and Heidelberg University were the most influential institutions, with total link strengths of 1,618 and 1,540, respectively.

A total of 6,273 authors were identified as contributors to research on free flap procedures in plastic surgery. Among them, KNESER U (32 publications) and HORCH RE (17 publications) were the most prolific. To evaluate academic impact, the H-index—a metric that reflects both productivity and citation impact—was used to identify the top 10 most influential authors (15). These authors are visualized in Figure 4C, and their H-index values are listed in Table 2. Their publication records and citation metrics underscore their significant academic influence in the field.

**Table 2:** Top 10 contributing authors.

Rank	Authors	Counts	H-index	Co-cited Authors	Citations	Total link strength
1	Kneser U	32	11	KoshiimA, I	172	3,081
2	Horch RE	17	11	Wei, FC	122	2,598
3	Gazyakan E	12	3	Hidalgo, DA	105	1,649
4	Seikaly H	12	6	Cordeiro, PG	100	1,281
5	Vogt PM	12	7	Taylor, GI	99	1,765
6	Hirche C	12	7	Fischer, JP	90	1,772
7	Beier JP	11	8	Selber, JC	89	1,407
8	Dragu A	9	7	Kroll, SS	89	1,359
9	Giovanoli P	9	6	U Rken, ML	89	1,106
10	Kremer T	9	6	Brown, JS	81	767

Author co-citation analysis, which identifies authors cited together in the same reference list, is depicted in Figure 4D. KOSHIMA I (172 citations) and WEI FC (122 citations) were the most frequently co-cited authors. This analysis illustrates the intellectual structure and scholarly interconnectedness of the research community focused on free flap reconstruction.

### 3.4 Journals and co-cited references analysis

**Figure 5:**
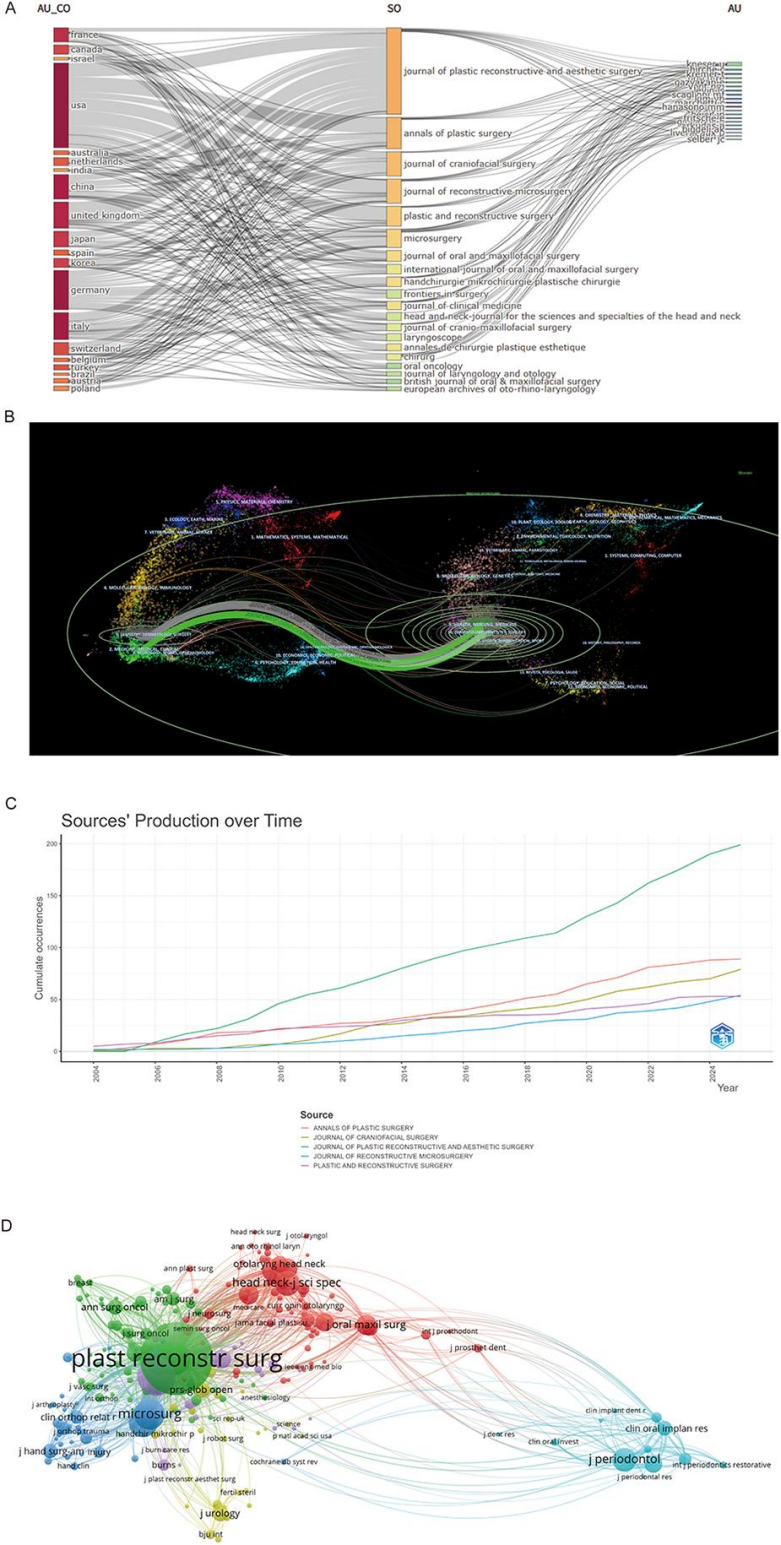
**(A)** Three-fields plot. Network relationships among countries, sources, and authors. **(B)** The dual-map overlay of journals. On the left were the citing journals (orange), on the right were the cited journals(blue), and the coloured path represented the citation relationship. **(C)** Top 5 productive journals dynamic publications analysis. **(D)** co-cited academic journals.

As shown in the three-field plot (Figure 5A), the United States leads in research output, with its contributions broadly distributed across the top 10 journals. American scholars predominantly publish in Annals of Plastic Surgery and Journal of Plastic, Reconstructive & Aesthetic Surgery, the latter also receiving substantial input from the United Kingdom and exerting considerable disciplinary influence.

A dual-map overlay of journals (Figure 5B) illustrates citation trajectories across research fields. Colored paths trace citation flows, primarily along two routes: from HEALTH, NURSING, MEDICINE and DERMATOLOGY, DENTISTRY, SURGERY to MEDICINE, MEDICAL, CLINICAL and NEUROLOGY, SPORTS, OPHTHALMOLOGY; and within the originating domains themselves.

Table 3 ranks the top 10 journals by citation frequency and publication volume. According to Journal Citation Reports (JCR), several are Q1 journals, including Plastic and Reconstructive Surgery, Head and Neck, Journal of Plastic Reconstructive and Aesthetic Surgery, Journal of Clinical Medicine, and Journal of Periodontology. Among them, Journal of Plastic Reconstructive and Aesthetic Surgery leads in both publication count (199 articles) and citations (6,423), underscoring its prominence. Annals of Plastic Surgery follows with 89 publications. Figure 5C presents publication trends in the top five journals, highlighting their growing influence.

**Table 3:** Top 10 journals and local-cited journals.

No.	Journal	Count	Impact factor (2024)	JCR partition (2024)	No.	Cited journal	Citation	Impact factor (2024)	JCR partition (2024)
1	Journal of Plastic Reconstructive and Aesthetic Surgery	199	2.4	Q1	1	Plastic and Reconstructive Surgery	6,423	3.4	Q1
2	Annals of Plastic Surgery	89	1.6	Q2	2	Annals of Plastic Surgery	1,796	1.6	Q2
3	Journal of Craniofacial Surgery	79	1	Q3	3	Journal of Plastic Reconstructive and Aesthetic Surgery	1,341	2.4	Q1
4	Journal of Reconstructive Microsurgery	54	2.3	Q2	4	Microsurgery	1,156	1.7	Q2
5	Plastic and Reconstructive Surgery	53	3.4	Q1	5	Journal of Reconstructive Microsurgery	1,047	2.3	Q2
6	Microsurgery	39	1.7	Q2	6	British Journal of Plastic Surgery (Continued as Journal of Plastic, Reconstructive & Aesthetic Surgery)	956	/	/
7	Journal of Oral and Maxillofacial Surgery	23	2.6	Q2	7	Head And Neck-journal For The Sciences And Specialties Of The Head And Neck	757	2.2	Q1
8	Handchirurgie · Mikrochirurgie · Plastische Chirurgie	22	0.6	Q4	8	Journal of Periodontology	678	3.8	Q1
9	Journal of Clinical Medicine	21	2.9	Q1	9	Laryngoscope	637	2	Q2
10	Annales de Chirurgie Plastique Esthétique	19	0.5	Q4	10	Journal of Oral and Maxillofacial Surgery	518	2.6	Q2

Co-citation analysis revealed 31,657 entities, forming six major clusters (Figure 5D). Plastic and Reconstructive Surgery had the strongest link strength (148,591), reflecting close collaboration with Annals of Plastic Surgery and Journal of Plastic Reconstructive and Aesthetic Surgery.

### 3.5 Citation and co-citation analyses

**Figure 6:**
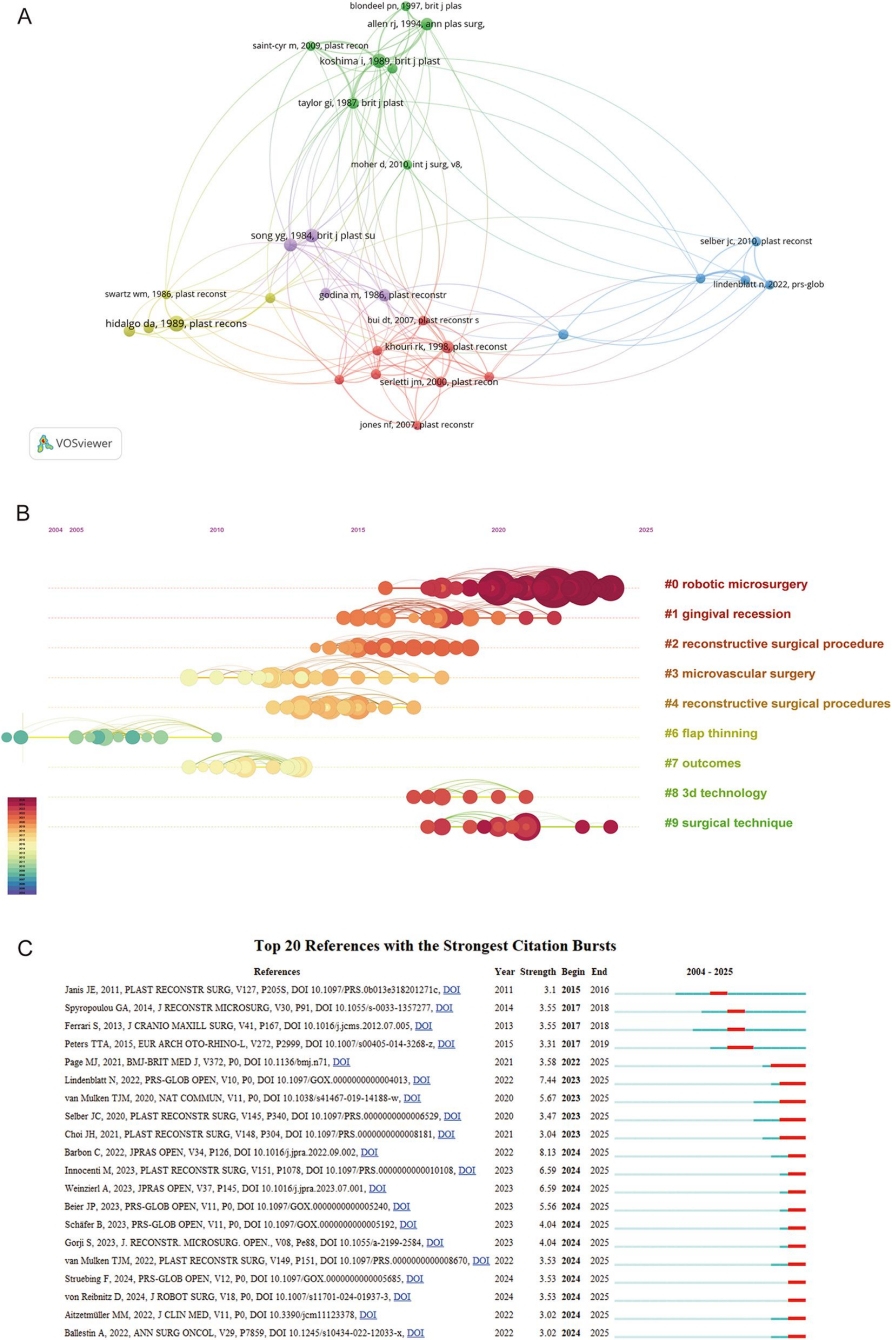
**(A)** Cluster analysis of co-cited references. **(B)** Timeline visualization of co-cited references cluster analysis. The size of each node corresponds to the number of co-citations for the respective journals, while the connecting curves between nodes signify co-citation relationships. The color on the right, red, is closer to the present, and purple is older. **(C)** The top 20 references exhibit the most pronounced co-citation bursts. The strength of a citation burst is a metric that quantifies the intensity of the burst during the period in which it occurs. Higher strength values indicate a more significant increase in citations over a short period, suggesting greater impact or influence in the field during that time.

Citation and co-citation analyses offer critical insights into the foundational literature of a research field (16). A reference co-citation network was constructed, as shown in Figure 6A, highlighting the seminal works of Hidalgo DA (17), Koshima I (18), and Song (19) as the most influential. Notably, these cornerstone studies represent distinct publication types: a case report, a research article, and a review, respectively (Table 4).

**Table 4:** Top 10 co-cited references.

NO.	Title	Citations	DOI	Type
1	Hidalgo DA, 1989, Plast Reconstr Surg	44	10.1097/00006534-198907000-00014	Case Reports
2	Koshima I, 1989, Brit J Plast Surg	34	10.1016/0007-1226 (89)90075-1	Case Reports
3	Song YG, 1984, Brit J Plast Surg	32	10.1016/0007-1226 (84)90002-x	Review
4	Wei FC, 2002, Plast Reconstr Surg	30	10.1097/00006534-200206000-00007	Article
5	Allen RJ, 1994, Ann Plas Surg	28	10.1097/00000637-199401000-00007	Case Reports
6	Godina M, 1986, PLAST Reconstr surg	27	10.1097/00006534-198609000-00001	Article
7	Khouri RK, 1998, Plast Reconstr Surg	27	10.1097/00006534-199809030-00015	Multicenter Study
8	Taylor GI, 1987, Brit J Plast Surg	23	10.1016/0007-1226 (87)90185-8	Article
9	Brown JS, 2010, Lancet Oncol	22	10.1016/S1470-2045 (10)70113-3	Review
10	Serletti JM, 2000, Plast Reconstr Surg	21	10.1097/00006534-200007000-00012	Article

The timeline view in Figure 6B illustrates the evolutionary path of research in this domain. Clustering analysis of cited references indicates two primary research hotspots in the application of free flaps in plastic surgery. The first centers on novel clinical indications—such as gingival recession—reflecting the expanding scope of free flap procedures. The second involves technological and procedural advancements aimed at improving flap survival and aesthetic outcomes. These include refinements to established techniques, such as microvascular surgery and flap thinning (20), as well as the integration of emerging technologies like robotic microsurgery and 3D printing, which further enhance the precision and efficacy of flap transplantation (Figure 6C).

### 3.6 Keywords co-occurrence and frequency

**Figure 7:**
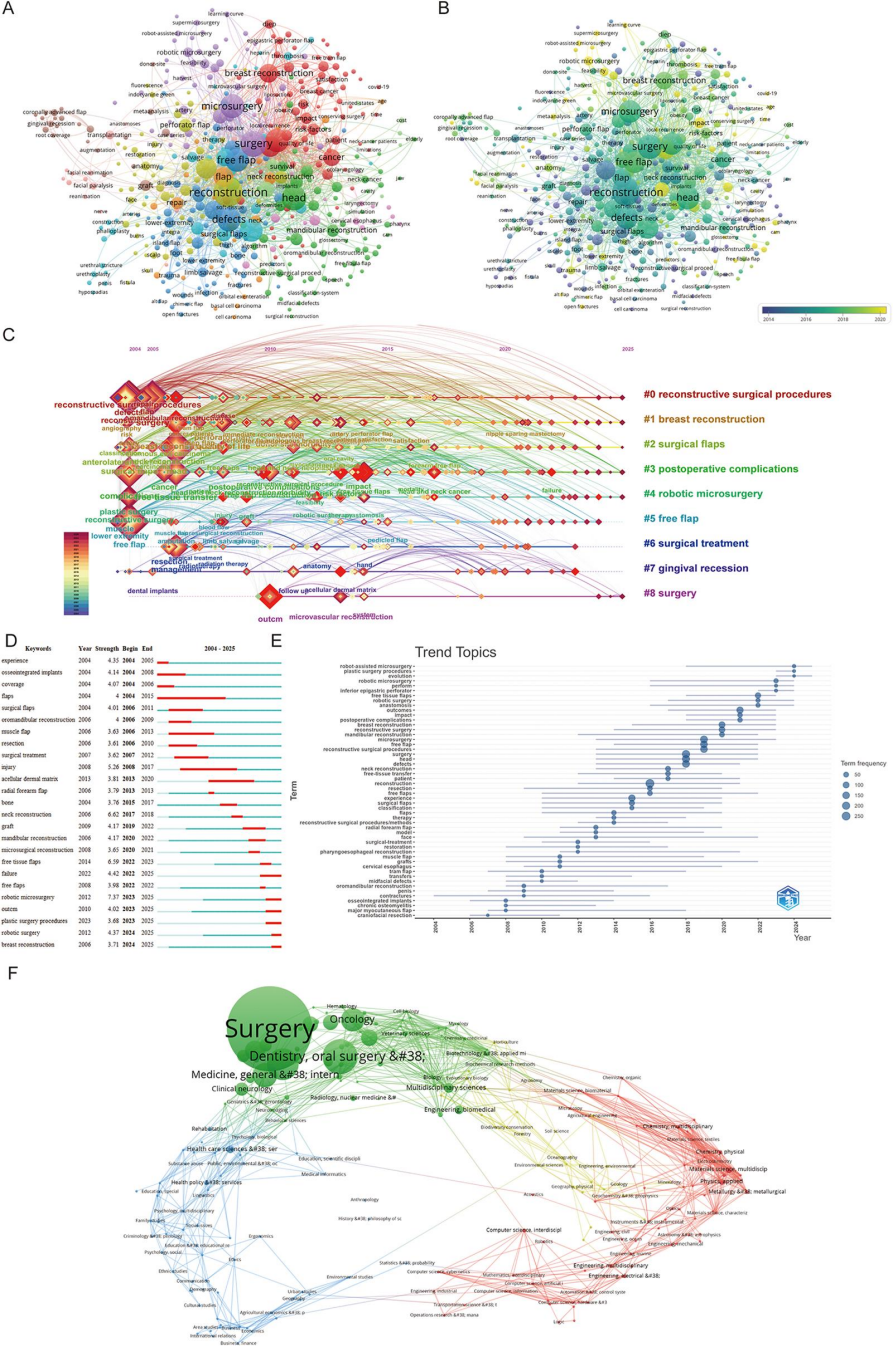
**(A)** Distribution of keywords according to the mean frequency of appearance. **(B)** Distribution of keywords according to the mean frequency of appearance. Keywords in purple appeared earlier. **(C)** Citespace keyword cluster timeline view. The right side of the image represents the names of the different clusters, the color of the light wheel reacts to the duration of the appearance of the research keywords, and the timeline with the connecting line reacts to the change of the keywords over time. **(D)** The frequency analysis and burstness of keywords topics terms. **(E)** The top 20 most cited keywords. The blue section represents the years from 2004 to 2025, and the red section represents the start and end years. Keywords with red lines extending to the latest year can indicate the research frontiers in a short period of time in the future. **(F)** Overlay of literature related to free flaps in the field of Plastic Surgery Procedures.

Visual keyword analysis enables the identification of key clinical focus areas in the application of free flaps within plastic surgery. In the co-occurrence map (Figure 7A), core terms such as “*reconstruction,” “complication,”* and “*microsurgery”* emerge prominently, reflecting the central themes in this field. Keyword clusters are organized into distinct thematic groups. The dark blue and light blue clusters primarily represent mature clinical applications, such as managing complex limb trauma caused by open fractures and procedures like urethroplasty. The temporal sequence shown in the timeline map (Figure 7B) further supports the maturity of these themes.

By contrast, the green and red clusters denote rapidly evolving research areas, including reconstruction of the head, face, and neck, as well as post-oncologic or post-mastectomy chest wall and local soft tissue reconstruction. The purple cluster highlights recent technological innovations, such as robotic-assisted microsurgery, which is receiving growing attention. These developments are further validated by their temporal progression in Figure 7B.

To provide a longitudinal perspective, we constructed a keyword cluster timeline visualization (Figure 7C). After excluding broad terms like “*free flap,”* the visualization still emphasizes two major thematic axes: clinical applications and technological advancements. The clinical axis includes terms such as “*breast reconstruction”* and “*gingival recession,”* while the technological axis features innovations like “*robotic microsurgery.”* In addition, keywords related to postoperative complications and aesthetic outcomes underscore an increasing emphasis on patient-centered care in reconstructive surgery.

A keyword burst analysis (Figures 7D,E) further illustrates emerging research hotspots and their respective timeframes. Early interest in “*muscle flap”* (2006–2013) highlights the clinical focus during that period on muscle-based flap techniques. More recently, terms such as “*neck reconstruction”* (2017–2018), “*mandibular reconstruction”* (2020–2022), and “*breast reconstruction”* (2024–2025) have gained traction. Notably, “*robotic microsurgery”* (2023–2025) has emerged as a significant focus area, consistent with the growing integration of advanced technologies into surgical practice.

To further examine the disciplinary breadth of this research, a domain-based overlay analysis was conducted (Figure 7F). Among the 1,407 articles analyzed, 65 unique subject areas were identified. *Surgery* emerged as the dominant category (*n* = 921), followed by *Dentistry, Oral Surgery & Medicine* (*n* = 157), *Otorhinolaryngology* (*n* = 129), and *Oncology* (*n* = 71). Forensic-facial reconstruction and post-oncologic repair remain core applications of free flaps. Notably, the overlay map reveals significant intersections between medical disciplines and technology-driven fields: *Multidisciplinary Sciences* and *Biomedical Engineering* (green cluster), *Computer Science* and *Robotics* (red cluster), and *Health Care Sciences* (blue cluster). These intersections suggest an encouraging trend toward cross-disciplinary integration, pointing to a future where industrial technologies play an increasing role in surgical innovation.

## 4 Discussion

### 4.1 Overview of the global quality and status of publications in this field

Over the past two decades, both the variety of free flaps and the scope of research on their clinical applications have expanded significantly. Notably, global research on free flaps for plastic reconstruction has surged, especially between 2019 and 2022, a trend likely driven by the increasing prevalence of cancer.

In terms of publication output, the United States leads, nearly doubling the output of Germany, and excels in total citations while maintaining a high average citation per paper. This reflects the substantial academic impact and high quality of its research. China has demonstrated impressive research momentum, surpassing previous leaders such as Germany and the United Kingdom in growth rate. Both the U.S. and China exhibit robust national collaborations, with the U.S. also showcasing a strong institutional presence, though Germany remains notable for its contributions. South Korea, China, and the United Kingdom, despite their significant research outputs, still face challenges in enhancing their institutional influence.

Among the 6,273 researchers, *Kneser U* and *Horch RE* stand out with the highest research outputs, H-index, and g-index, guiding future research directions. The co-authorship network reveals a strong preference for domestic collaboration, highlighting the need for more international partnerships to foster knowledge exchange, resource sharing, and innovation.

Regarding journal publications, *The Journal of Plastic, Reconstructive & Aesthetic Surgery* and *The Annals of Plastic Surgery* lead in article volume, with publications increasing annually. The U.S. and the U.K. contribute the highest number of articles. Despite fewer publications, *Plastic and Reconstructive Surgery*—a Q1 journal—holds the highest local citations and co-citations, underscoring its significant influence and reputation.

### 4.2 Hotspot analysis of research

The results from keyword extraction and analysis indicate that free flaps are a current research hotspot in the field of plastic surgery, reflecting the latest trends in the field. Current studies predominantly fall into two main categories: the practical clinical applications of free flaps and the technologies related to free flaps.

#### 4.2.1 The practical clinical applications of free flaps

Our analysis reveals that free flap procedures were adopted promptly and have reached a high level of maturity in treating open tibial fractures and urethral reconstructions.

##### 4.2.1.1 Early adoption (1980s–1990s)

Free flaps were rapidly integrated for complex cases, enabling one-stage penile reconstructions since 1984 (21) and advancing urethroplasty/post-cancer aesthetics (22). By 1997, extensions to open tibial fractures reduced healing times by ∼30% vs. local fasciocutaneous/trapezoid flaps (23), with innovations like antibiotic cement-coated plates elevating survival rates to >95% (24). Prevalence rose from ∼20% to 60% during this era (per procedural trends).

##### 4.2.1.2 Contemporary dominance (2000s–present)

Focus has pivoted to post-tumor reconstructions, with breast and head/neck cancers leading. Breast cancer—prevalent in women and often yielding suboptimal aesthetics post-excision (25)—now favors the DIEP flap for reconstruction, achieving >90% patient satisfaction and validated safety in meta-analyses for volume enhancement (26, 27). In head/neck tumor resections, which disrupt speech/mastication/swallowing and facial aesthetics (28), free fibular flaps (FFF) are now gold standard for restoration. However, FFF's thicker skin risks inflammation/pain/bleeding (29), mitigated by prompt revascularization; ossification mismatches challenge dental implants (30), necessitating customized designs. Overall, this era marks free flaps' maturation as cornerstone techniques.

#### 4.2.2 The related technologies of free flaps

Free flaps are evolving toward aesthetic and minimally invasive approaches (31). This field has become a literature-highlighted multidisciplinary nexus, integrating computer science and mathematical design to model and monitor free flaps, thereby minimizing complications. Literature trends show a surge in publications on 3D printing, computer-aided design (CAD), and computer-aided manufacturing (CAM) since the mid-2010s. Recent advancements have made significant progress in preoperative reconstruction, improve the precision of osteotomies and reconstructions. This method not only facilitates free flap grafting but also establishes a foundation for enhanced surgical outcomes (32). For instance, a 2022 retrospective study by Donald J. Annino Jr. et al. (33) reported on 26 cases of mandibular reconstruction using free flaps guided by virtual surgical planning (VSP) and 3D-printed templates. Intraoperatively, only two cases required minor adjustments, and there were no instances of flap necrosis. Among the patients, 20 out of 21 (95%) experienced pain relief, 13 out of 20 (65%) showed improvement in trismus, and 21 out of 24 (87%) achieved correction of preoperative malocclusion or jaw deformity. In addition, a randomized controlled trial investigating aesthetic reconstruction of maxillary defects using free scapular flaps combined with CAD/CAM-customized osteotomies (34) demonstrated significant improvements in facial symmetry. These improvements can be attributed to enhanced osteotomy precision and reduced ischemia time, ultimately contributing to better long-term flap survival and functional outcomes (35). This reflects the technology's growing representation in literature, from pilot cases to validated outcomes.

The integration of augmented reality (AR) and virtual reality (VR) technologies into surgical planning has markedly enhanced the precision and efficiency of free flap procedures. AR enables surgeons to overlay preoperative imaging directly onto the patient's anatomy, facilitating highly accurate localization of perforator vessels—an essential determinant of flap viability (33, 36, 37). A 2025 pilot study employing AR for preoperative perforator mapping in anterolateral thigh (ALT) flap reconstruction reported a flap survival rate of 95.8%, with only one case (4.2%) of partial necrosis among 24 patients. The mean discrepancy between actual and AR-identified perforator locations was 3.54 ± 2.80 mm (95% CI: 2.58–4.50), which was significantly lower than that observed with conventional color Doppler ultrasound (9.57 ± 5.84 mm; 95% CI: 7.75–11.58; *P* < 0.001) (38). Literature evolution underscores AR's superiority, with publications shifting from 2020s comparisons to 2025 systematic validations. Furthermore, a 2025 systematic review confirmed the superiority of AR over Doppler ultrasound in perforator identification, demonstrating greater accuracy during flap planning and dissection, along with a substantially shorter time required for flap harvest (39). While VR has traditionally required the involvement of biomedical engineers for implementation, recent innovations have improved its accessibility. A recent case report demonstrated that novel VR software now enables surgeons to independently complete preoperative planning for fibula flap mandibular reconstruction in under five minutes—without the need for technical support (40). This advancement has significantly increased the accessibility and practicality of VR technology in clinical settings. These technological developments not only reduce the incidence of complications such as flap necrosis but also streamline the surgical workflow, reinforcing the status of AR and VR as indispensable tools in modern reconstructive surgery. Their prevalence in literature has grown exponentially since 2020, particularly for orbital and mandibular defects, mirroring broader free flap trends.

Based on the clustering analysis discussed earlier, robotic microsurgery has emerged as a prominent research focus. Robotic systems offer enhanced dexterity, elimination of physiological tremor, and superior three-dimensional visualization (41), aligning well with the sub-millimeter precision required for microvascular anastomoses in free flap reconstruction. Although widely adopted in urology and gynecology, the application of robotic systems in plastic surgery remains underexplored. However, growing has spurred development of dedicated robotic platforms such as the Symani Surgical System (42) and MUSA (MicroSure) (43), tailored to the specific demands of microsurgical procedures. A pivotal 2023 study (44) described 23 free flap reconstructions performed using the Symani system, including 11 radial forearm flaps, 7 ALT flaps, 4 fibula flaps, and 1 serratus anterior flap. The study demonstrated the feasibility of performing both end-to-end and end-to-side arterial and venous anastomoses with robotic assistance. Clinical applications of robotic-assisted free flap reconstruction have garnered increasing attention in recent years. For instance, a prospective study by Selber et al. validated the safety and efficacy of robotic-assisted latissimus dorsi free flap harvests, showing reduced donor site pain and decreased risk of abdominal wall herniation or bulging (45). These applications are being extended to head and neck reconstruction, potentially offering improved aesthetic outcomes, shorter operative times, and fewer complications (46, 47). Nonetheless, a literature review identified several challenges associated with robotic free flap procedures, including the lack of haptic feedback and reduced efficiency in anastomosing large vessels such as the internal jugular vein (48). Additionally, robotic microsurgical systems present a steep learning curve, necessitating extensive training and practice (49). Despite these limitations, the precision offered by robotic microsurgery holds significant promise for improving surgical outcomes. We believe that with continued technological refinement and surgical adoption, robotic systems are well-positioned to become standard tools in the field of plastic and reconstructive surgery.

Research in materials science is closely linked to surgical outcomes and the durability of postoperative flaps. There have been efforts to advance the conventional reconstructive ladder by incorporating regenerative medicine principles, aimed at enhancing flap reconstruction (50). This involves leveraging the regenerative capabilities of cells and tissues *in vivo* through the application of biomaterials and specific biochemical stimuli. Notably, the use of 3D bioprinted biomaterials has been shown to improve the survival of free flaps by creating inorganic or synthetic polymer structures that facilitate bone repair and provide a foundation for flap grafting (51). Moreover, 3D bioprinting techniques that produce hydrogels not only aid in bone regeneration but also enhance flap survival (52), contribute to anti-infection measures (53), and promote neovascularization within the flap (54–56). Additionally, challenges such as muscle volume loss post-flap transfer can be addressed through the use of bionic scaffolds composed of natural and synthetic hydrogels, which support cellular growth and differentiation, thereby enhancing regenerative outcomes. Furthermore, cellular products like platelet-rich plasma (57) and exosomes (58) have been identified as promising agents for improving immunoprotection and anti-inflammatory responses, highlighting their potential for future research and development. Overall, materials science literature has evolved from basic biomaterials (2010s) to integrated bioprinting and cellular therapies (2020s+), increasingly represented in free flap studies for complication reduction.

### 4.3 Limitation

Our study possesses several limitations. Firstly, by selecting only the WOSCC database, Pubmed database and Embase databases, we cannot ensure comprehensive coverage of literature on free flaps within plastic surgery. Secondly, we restricted our analysis to literature written in English, which introduces a potential language bias.

## Data Availability

The original contributions presented in the study are included in the article/Supplementary Material, further inquiries can be directed to the corresponding authors.

## Supplementary material

Supplementary Figure S1 Search strategy.
