# Supplementary figures and images for: Clinical application and developmental direction of free flaps in plastic and reconstructive surgery procedures: a bibliometric analysis
Source: Front Surg. 2025 Nov 19;12:1661571. doi: 10.3389/fsurg.2025.1661571 (PMC12672497; doi:10.3389/fsurg.2025.1661571)

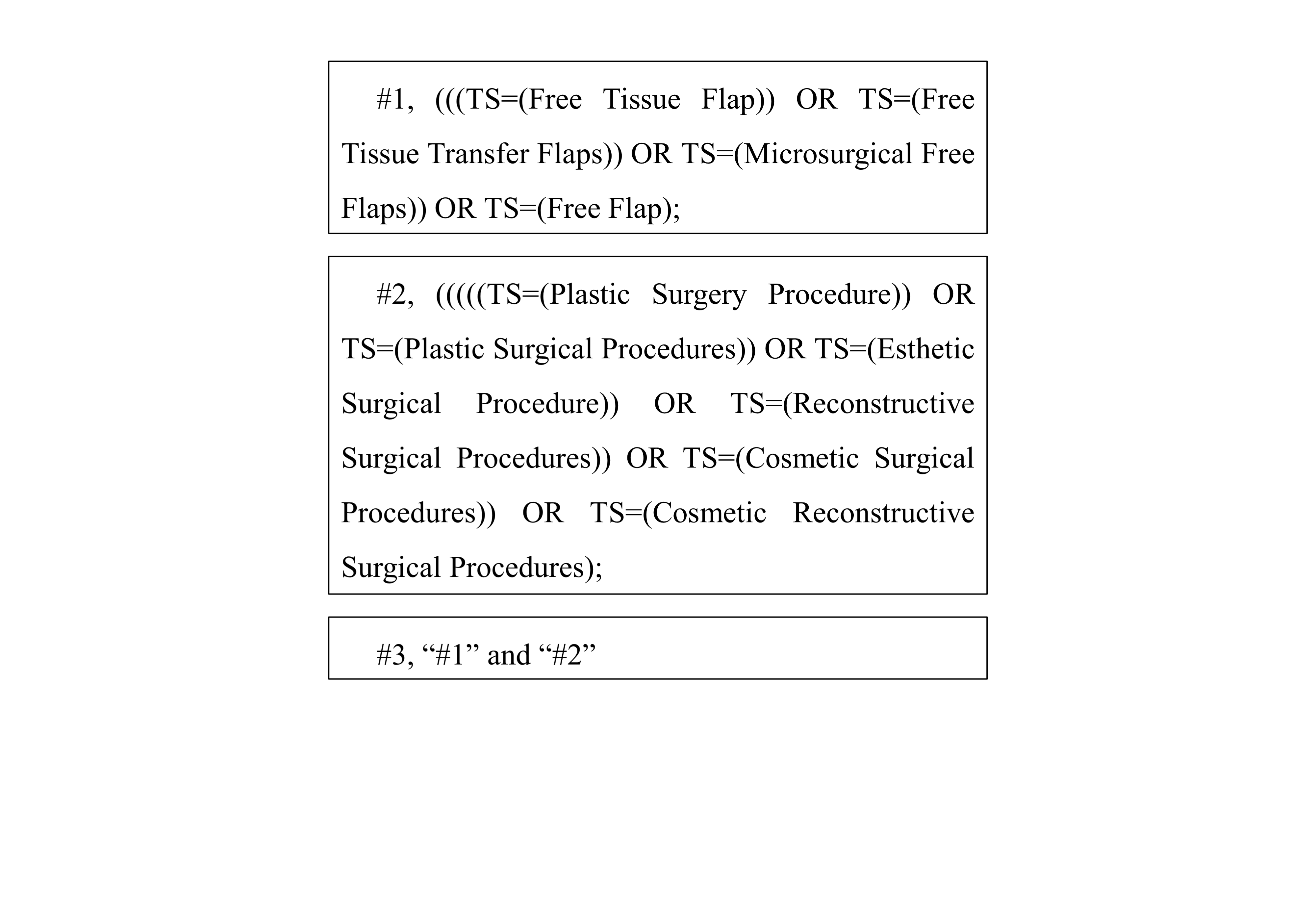

Supplement: Supplementary Figure S1 — Search strategy. [file Image1.tif]
